# There is No Supporting Evidence for a Far Transfer of General Perceptual or Cognitive Training to Sports Performance

**DOI:** 10.1007/s40279-024-02060-x

**Published:** 2024-06-21

**Authors:** Job Fransen

**Affiliations:** School of Allied Health, Exercise and Sports Sciences, Charles Sturt University, Building 801, Lake Innes Road, Port Macquarie, NSW 2444 Australia

## Abstract

In this opinion piece I reiterate the concepts of near and far transfer as previously described in the psychological literature. I show that despite very limited evidence, many technologies, tools and methods make questionable claims of eliciting far transfer from generic perceptual and/or cognitive training to sports performance. Specifically, this commentary illustrates with studies on stroboscopic vision, neurofeedback training and executive functions that the claims made for the beneficial effects of these training methods are currently unsubstantiated. I conclude that greater scrutiny by researchers is needed in order to assist practitioners to make better-informed decisions about tools, methods and technologies that may aid sports performance.

## Introduction

Perceptual and/or cognitive training (PCT)—in one form or another—is common in sports practice [1]. There are generally two different types of PCT considered in the literature: specific or generic [2, 3]. Specific PCT focuses on perceptual and/or cognitive processes in contexts that closely align with those in the target setting (i.e. the task for which training aims to prepare the athlete). An example of such a method would be neurofeedback training, where athletes are taught to generate specific brain activity considered to be beneficial for performance, during skill performance. Generic PCT, on the other hand, usually targets perceptual and/or cognitive abilities in isolation (i.e. in a context that deviates from the application task). An example of generic cognitive training is the training of executive functions (i.e. higher level cognitive functions such as cognitive flexibility or response inhibition) through computerised tasks. It is important to note that while some PCT methods can be viewed as more cognitive (e.g. neurofeedback or executive function training) or perceptual (e.g. stroboscopic vision training), contemporary skill acquisition research argues that the perceptual and cognitive components of movement can hardly ever be truly separated from one another (e.g. Inns et al. [4]). This is clear throughout the literature where, depending on the study, specific training tools such as video-based anticipatory training are viewed as either cognitive training [5] or PCT [6]. Hence, in the context of this current opinion article, the term PCT will be used to describe any training that focuses on improving perceptual and/or cognitive processes.

Appelbaum and Erickson [2] discussed a variety of PCT tools and methods with a perceptual emphasis, such as low-level visual instruments, perceptual-cognitive training instruments, visual-motor reaction training, integrated sensorimotor training tools, stroboscopic vision training, eye tracking and quiet eye training, and sports simulations and virtual reality training, each with a differing degree of specificity to sports performance [2]. This variety in PCT tools is echoed in a recent scoping review by Choo et al. [7], who revealed that there are 19 randomised controlled trials (RCTs) in the skill acquisition literature using perceptual-cognitive training interventions. These interventions span those that can be considered more specific (e.g. virtual reality simulations, visual occlusion paradigms, quiet eye training) as well as generic (vision training, video-based perceptual training or training altering attentional processes) training. Walton and colleagues [5], in a perspectives article on the potential role for cognitive training in sport, list a number of PCT methods which they consider to have a more cognitive focus, such as computerised cognitive training, video-based anticipation training, above real-time decision-making training, among others.

While it is obvious that PCT are commonly used and reported in the sports science literature, the evidence that supports whether PCT transfers from training to sport performance has thus far been limited [3, 6, 8]. Therefore, this current opinion article serves as a timely reminder and refresher of the need to carefully consider the usefulness of PCT tools in sport that claim to be able to influence sport performance. Hence, this current opinion article aims to first (re)introduce the reader to the concepts of near and far transfer, before addressing some of the evidence for the existence of near and far transfers from PCT within and outside of the sporting domain in order to caution practitioners and researchers about claims made for far transfers from PCT to sports performance in the sports science literature or by technology companies providing PCT tools or methods.

### A Brief Introduction to Near and Far Transfer

A transfer of skills is the generalisation of skills that are acquired through training across different domains. A near transfer occurs when the training context and the trained behaviour are very similar to the application context [9] and application behaviour, for example when a coaching team uses video-review sessions to improve athletes’ knowledge of the game so they become better at applying that knowledge in similar video-review sessions in the future. In contrast, far transfer occurs when the training context and the trained behaviour are dissimilar to the application context and application behaviour [9], for example when coaches engage in video-review sessions with the goal of improving on-field performance. For a detailed perspective on, and more examples of, near and far transfer, consult Sala et al. [9]. In the psychological literature, it is well understood that near transfers are very common, while far transfers, though much more interesting to study or achieve, are very rare [10]. This is in a sense logical. We can expect, for example, that the training of juggling skills makes you a better juggler but may not have any influence on your ability to balance on one foot, given how little one task resembles the other.

However, researchers, consultants, and external service providers rarely make an interesting business or research case facilitating near transfer. As a result, many make claims their technologies or methods can facilitate far transfer. In other words, using their cognitive training tool or method will make you a better penalty taker, esports player and/or referee, and more. But what really is the scientific basis for these claims? Should each and every one of these tools, methods and claims be evaluated individually, or does evidence already exist that can help organisations make sense of whether the cognitive training tool they are being offered has any chance of living up to the far transfer promises made by the claimants?

To build the case for this current opinion article, a piece of high-level evidence from the psychological literature will be introduced first to illustrate the lack of empirical support for far transfer claims in the cognitive training literature in psychology, before providing some specific examples of cognitive training tools which are often used to facilitate far transfer to sporting performance.

### There is No Supporting Evidence for Far Transfer Claims of Cognitive Training in the Psychological Literature

The authors of a meta-analysis of meta-analyses [9] (a powerful method that combines the evidence reported in many meta-analytical studies in a defined area to see if their claims can truly be substantiated, and as such provides a high level of evidence) first examined what the evidence was for a near transfer of working memory training (a common form of cognitive training) to other related memory tasks across a variety of samples (including both healthy and non-healthy children and adults). In the second part of their review, they examined the evidence for a far transfer from working memory training to other cognitive domains such as fluid reasoning, cognitive control, language etc. The findings of this second-order meta-analysis are briefly summarised here and serve as a contemporary status quo of the psychological literature on the near and far transfer of cognitive training and offer a (re) introduction of the current body of evidence related to the existence of near and far transfers from cognitive training in a research area related to sports science (i.e. psychology).

First, to study the near transfer of working memory training to other, related memory domains, four meta-analyses were included in the second order meta-analysis by Sala et al. [9] using samples of typically developing children, children with learning disabilities, healthy adults and adults with some level of mild cognitive impairment. The authors concluded that working memory training is generally related to better performance in subsequent memory tasks, especially in typically developing children (this same effect also exists for both adult groups but is less clear for children with learning difficulties). In conclusion, working memory training transfers to memory performance. Then, to investigate far transfer between memory training and other cognitive assessments, the authors used the same method but altered the outcome variables of interest. Rather than examining the effect of working memory training on memory tasks, they examined its effect on other higher-level cognitive functions such as fluid reasoning, cognitive control, processing speed and language. The researchers found no evidence that far transfer occurred between working memory training and these cognitive tasks.

From this second-order meta-analysis it can be concluded that within the psychological literature, there is (i) evidence for near transfer from working memory training to memory tasks, but (ii) no supporting body of evidence for a far transfer to other types of high-level cognitive functioning. Furthermore, the authors also included an analysis of meta-analyses which include far transfer results from other forms of cognitive training such as video gaming, exergaming, music training and chess training and concluded that these types of ‘alternative cognitive training’ yielded zero results in terms of their far transfer to other, unrelated cognitive skills. As a result, they concluded that support for a near transfer of cognitive training exists and is likely modulated by the characteristics of the trainee, yet they did *not* find support for a far transfer of various modalities of cognitive training.

While this study by Sala et al. [9] offers valuable insights into the current state of the memory training literature, its findings are not unexpected or novel. Previously, Simons and colleagues [11] concluded that there is extensive evidence that brain-training interventions improve the trained behaviour (i.e. near transfer), yet there is far less evidence that these interventions improve closely related tasks or everyday cognitive performance (i.e. far transfer). Similar conclusions have been drawn from research in sport where Harris et al. [8] found no supporting evidence of a far transfer of cognitive training to sports performance in a systematic review of the literature.

### What About Far Transfers of Perceptual and/or Cognitive Training in Sport?

Several studies have addressed the relationship between perceptual and cognitive training and sport performance. While a discussion of an exhaustive list of all studies is outside of the scope of this opinion piece, I highlight two relatively recent publications reviewing the current literature with regards to far transfer effects of CPT in sport. The first systematically reviews currently available commercial cognitive training devices [8], while the second offers a narrative review, albeit using a systematic search of the literature [12], which critically addresses evidence for far transfer of vision training to sport performance.

The systematic review by Harris and colleagues [8] searched the available literature for intervention studies using commercially available cognitive training tools applied to adult participants and which assessed either near or far transfer. They found that while all studies included measures of near transfer, about half of the retained studies in the review assessed some aspect of far transfer (i.e. a measure other than a cognitive test), yet the majority of those were self-report measures. Upon further analysis, only four of the studies in the systematic review included a far transfer test that could be considered representative of real-world performance, and only one of those [13] assessed far transfer to a sports-related task (i.e. coach ratings of passing accuracy). This study found that training with Neurotracker in nine experimental participants improved coach ratings of passing accuracy, but not dribbling or shooting accuracy, compared with seven active controls. Hence, this study [13] concluded somewhat prematurely given its methodological limitations (e.g. small sample size, bias introduced by the fact that subjects all originated from the same football team and the lack of participant or researcher blinding, among others), that it provided the first evidence for the far transfer effects of cognitive training to on-field performance. Harris and colleagues recognised these methodological shortcomings and concluded in their review that many of these commercial cognitive training tools do indeed elicit near transfer to the trained cognitive ability but that “the evidence provides little indication that commercial cognitive training devices can transfer to the sports field” [8, p15].

In the second review on vision training and on-field performance, Laby and Appelbaum [12] critically reviewed the empirical literature linking visual assessment and/or training to athletic performance. In their review, which used a systematic search process of the literature followed by a narrative review, the authors reviewed a subsample of what they considered to be 16 articles addressing relationships between visual training and game or game-like statistics or benchmarks of performance which are representative of the broader literature. The studies included in this review were heterogeneous in their research questions, methodologies, findings and research quality. Many of the studies examined in this review showed (some) positive relationships between cognitive training and sports-related performance (i.e. baseball batting, video-based decision making in badminton, coach-rated passing accuracy, etc.), yet the authors of this review [12] report that their methodological shortcomings such as the low statistical power, the frequent lack of control groups and/or randomisation and the use of non-representative outcome measures make it difficult to draw consistent conclusions from the literature. Indeed, it would be problematic to draw causal relationships between vision training and sport performance based on these combined studies as underpinning evidence. One study [14] also reported in Laby and Appelbaum’s [12] review has attempted to address this by pre-registering their randomised control trial before the study was conducted, which is applauded by the reviewing authors. While this is indeed a step in the right direction, and offers the opportunity to more rigorously examine the effects of cognitive training interventions in empirical studies, the pre-registration does not preclude this study from suffering considerable risk of bias, especially when the pre-registration procedures and study procedures differ significantly. As a result, the recruitment of a convenience sample which may not represent the population of baseball players, the pseudo-randomisation process used, the significant drop out especially in the intermediate and far transfer measures, the unequal exposure to baseball in participants from different universities and the potential lack of blinding of those developing the training intervention introduce a significant risk of bias.

Having reviewed some of the empirical studies available on the far transfer potential of cognitive and/or perceptual training through two prominent reviews in the area, the conclusion remains that there is no consistent supporting evidence in favour of far transfer from PCT to sport performance.

## Why Do We Keep Investing in Tools That Make Exaggerated Claims?

The lack of supporting evidence for far transfers from PCT in the psychological and sports literature should at least make us question the usefulness of PCT for the improvement of sports performance. Yet, many technology companies and researchers continue to invest in training tools, methods and technologies that claim to facilitate far transfer. This current opinion piece offers several potential reasons for why this may occur which are partially informed by my own experiences.

1. The claims made by technology companies selling PCT are seemingly logical for those making purchasing decisions at sporting organisations. More often than not, an elevator pitch by one of these technology companies will start with: “A player needs to be able to make split-second decisions while on the pitch…”. This is often done to establish the role played by the brain in executing the right skill at the right time (this is even referred to as “game intelligence” in some instances). For many practitioners, this cements a logical causal relationship between improving the function of the brain and improvements in sports performance. Given that the relationship is so logical, and that most staff members of sporting organisations may find it difficult to think critically about these tools, purchasing decisions for these tools are often made without sufficient scrutiny of the claims made in support or PCT.
2. External pressures unrelated to whether PCT is effective or not drive purchasing decisions. As a consultant, much of my work consists of helping sporting organisations to scrutinise the claims made by technology companies who claim to have the next breakthrough training tool that will accelerate the development of athletes’ skill and decision making. Often, staff members or players who have seen these tools being used by their competitors or on social media (or worse, *by* their competitors *on* social media) are the first to bring this training tool to the attention of the sports science staff at sporting organisations. This may implicitly pressure them into consider these tools for performance improvement, regardless of whether evidence supporting their efficacy exists in the first place. In my experience, tools that have not received sufficient scrutiny and have been purchased through a fear of missing out are indeed acquired by the organisation and then briefly used before they are quickly discarded or set aside.
3. Research is too slow to catch up with new technological advancements. Many sport scientists contribute to the field by developing rigorous studies that test some of the most prominent, yet unsubstantiated claims made in the sports skill learning realm. Yet, it takes researchers several years to find financial support, design, implement and publish the findings of a study. By that time, the technologies have long been purchased and discarded (or worse, continue to be used without scientific scrutiny because a sporting organisation has now ‘bought into’ its use), and the scientific work of the researchers presenting doubts about the efficacy of the tool is lost to cognitive dissonance on the part of the purchaser.
4. Whether or not PCT is capable of eliciting a far transfer to sports performance may not play a role in purchasing decisions. Perhaps the reason why organisations actively invest in PCT is not to achieve a far transfer but to improve athletes’ engagement, motivation and drive to train, which may have indirect effects on performance. Hence, the belief in the effectiveness of PCT, and not the effectiveness of PCT in and of itself, may be the driver behind sporting organisations’ continued investments. One theory which may be relevant for readers of this article to explore is Expectancy Theory (e.g. Holton [15]), which argues that individuals are more likely to be motivated if they believe their training efforts will lead to improved performance.

## Examples of Tools and Methods With Claims of Far Transfer in Sport

### Stroboscopic Vision Training for Sports Performance

Popular media has previously reported on how stroboscopic vision glasses have been used by prominent sportspeople during practice (e.g. NBA basketballers [16], professional ice-hockey players [17, 18]). In fact, I even used them myself with the aim of improving sports performance while I worked at a prominent Australian sporting franchise [19]. Prominent sportspeople publicly using stroboscopic vision glasses to improve performance may have inadvertently acted as an endorsement of the claim that they improve performance. As is often the case, the claims made to support the efficacy of stroboscopic eyewear in the scientific literature are logical [20, 21]. When wearing these glasses, the amount of visual information available in the environment is greatly reduced, forcing the athlete wearing the glasses to extract and process relevant information in the environment quicker. As a result, the athlete is then able to perform better when not wearing the glasses, because they have become accustomed to needing to pick up and process information quicker. Indeed, studies that have examined the relationship between wearing stroboscopic glasses and sports performance inferred from their results that reducing how much visual information is available during a sport task impairs motor performance [22, 23], which substantiates the claims that stroboscopic vision training adds a level of difficulty to a task beyond that of full vision training.

Several studies have examined the effects of training with stroboscopic glasses on subsequent performance in perceptual (near transfer) and sport (far transfer) tasks. The findings of these studies largely align with what we already know about the prevalence of near and far transfers of skill from psychological research. There is substantive evidence to suggest training with stroboscopic glasses leads to a near transfer to perceptual-cognitive function (motion-sensitivity [24], short-term memory [25], anticipatory timing [26], visual acuity [27], and many more) yet the evidence for a far transfer to sport performance is inconclusive to say the least (e.g. in ice hockey [28] or badminton [29]), or not supportive of the existence of far transfers altogether (e.g. in football [20] or badminton [30]). As a result, while stroboscopic glasses could likely be used to increase the difficulty of practice sessions [23], there is no irrefutable evidence to suggest that training under intermittent visual restriction leads to a far transfer to sport performance. Carroll et al. [31] similarly concludes that research on stroboscopic vision interventions for optimising sports performance is affected by a huge variance in study designs, which limits the drawing of firm conclusions on sports vision training effectiveness for the development of sports skills.

### Neurofeedback Headsets in eSports

Neurofeedback training is a hot topic in neuroscience. Neurofeedback is a type of biofeedback training (for an overview of the effect of augmented feedback training on the performance and learning of sports-relevant skills, see Petancevski et al. [32]). The premise of neurofeedback training is again logical. Experts usually exhibit different brain activity than novices across a range of tasks, including during the learning of motor skills [33]. Therefore, practice that encourages athletes to have more expert-like brain activity may encourage learners to learn certain skills faster and as a result perform them better. Neurofeedback comprises specific practice methods in which learners are given feedback on their brain activity through an electroencephalogram, so they can learn to produce cortical activity patterns that usually belong to experts to speed up the learning or improve the performance of a task [33].

As a result of the logic behind neurofeedback training, many organisations have implemented neurofeedback training to improve motor performance in a variety of domains. One of the most prominent is esports. Esports or electronic sports is a form of competitive gaming. It is a rapidly growing domain of human–computer interaction whereby players manipulate virtual worlds to achieve a specific task goal (e.g. drive a virtual car presented on a screen using a controller). eSports competitions attract thousands of competitors and millions of spectators, and prize pools for those victorious are also significant as a result. Consequently, esports companies invest heavily in training tools, instruments and methods that can give them an edge over their rivals. One of the methods that has seen significant investment is the use of neurofeedback headsets or headbands.

Several neurofeedback training instruments such as ‘brainwave reading EEG headsets/ headbands’, ‘neurofeedback software’ and ‘brain-training wearables’ exist, yet their claims to “improve and optimise performance” cannot be substantiated by the existing body of literature. Several studies have examined if neurofeedback training can be used to improve sports performance. Most do conclude that neurofeedback training leads to a near transfer by teaching learners to change their cortical activity (e.g. reduce frontal high-alpha power [33]). However, there are several methodological issues that limit our understanding and implementation of neurofeedback training for sports performance (i.e. a far transfer [34]) such as low statistical power or the absence of placebo groups [35]. As a result, neurofeedback ‘wearables’ in esports (regardless of whether these are valid measurement or training instruments in the first place) do not (yet) succeed in providing evidence to back their claims of transferring learned behaviours during training to competition games.

### Developing Executive Functions for Football Expertise

Executive functions are a multifactorial control mechanism that modulates cognitive processes to regulate human cognition and goal-directed behaviour and have been proposed to play a role in domains such as self-regulation, childhood development, academic achievement and the development of expertise [36]. The study of executive functions in the context of sporting expertise is especially common in football [37]. Again, the argument here is sensible when we argue logically about how the need for proficient inhibitory control (a commonly reported executive function) logically relates to playing football at the highest level. A footballer who sees a clear and open passing line to a teammate in a position to score lines up a pass. However, a sudden movement from an opponent suddenly blocks the passing line. A good player then inhibits the initial passing action and perhaps chooses a different teammate to pass to or exploits the gap left by the moving opposition player to move closer to the goal. This seemingly logical reasoning would lead to the conclusion that a footballer with superior executive functions will perform better on the field.

Indeed, some studies have claimed that executive functions are related to football expertise. For example, Vestberg and colleagues [38] claim that executive functions were related to the number of goals and assists a player made in competition two years later. They concluded that the results of this study strongly suggest that results in cognitive function tests predict future success of footballers, which fuelled many of the investments made into cognitive assessment and development in football. However, Beavan and colleagues [37, 39–41], in a series of several studies conducted inside an organisation that implements cognitive assessment and training in high-level footballers, found no evidence to support the claim that executive functions are strongly related to football expertise. In fact, Beavan et al. [40] showed that the relationship between football and expertise is likely largely mediated by a participant’s age and even revealed that professionally contracted players showed poorer executive functions than players in late adolescence [40]. Furthermore, previous work has shown that executive function training (e.g. 3D motion tracking training in Scharfen and Memmert [42]) shows a near transfer to 3D motion tracking performance but very limited far transfer to other executive functions such as attention, working memory capacity, cognitive flexibility etc. As such, executive function training likely elicits a near transfer that is specific to the executive function being trained (i.e. players engaging in cognitive training improve their cognitive performance in related tasks) but likely does not yield a far transfer to football expertise or performance.

## Conclusion

This current opinion piece is a reaction to my increased involvement in requests from sporting organisations to evaluate the claims made by new technologies and the companies that use them, in which they claim that PCT can facilitate far skill transfer (i.e. the transfer from PCT to domain-specific sports performance). Psychological research has provided a sound evidence base to suggest that these far transfers are rare, and if a transfer of skills does occur, it is likely to be a near transfer from cognitive training to related cognitive skills. I have used three specific examples of PCT technologies or methods of which the effectiveness on the learning of sport skills hinges on a rare far transfer occurring. This current opinion piece provides arguments and examples to contend that these claims of far transfer are exaggerated. It is intended for this work to reach sport practitioners who regularly deal with the emergence of new technologies and similar methods, so that they can make better informed decisions about their application.

It is not the intent of this article to argue that near transfers cannot be useful. In fact, much of what is understood about training progression is based on our understanding and reliance on near transfer. For example, for an athlete to become stronger or return to play after injury, strength and conditioning coaches and rehabilitation specialists have long used a sequence of near transfers where each progression of a movement is built on a previous one with a slight adaptation to being more specific to the final application task of competitive performance. This may be true to the same extent here. PCT could be part of a long chain of near-transfer tasks, where the application task is the next step in the progression chain (for example, stroboscopic vision training may yield a near transfer to improved visual function such as motion sensitivity or anticipatory timing in a task slightly more specific to the application task which represents but one link in a much longer chain of sequenced near-transfer tasks connecting the initial training task to improved sports performance). Unfortunately, those wishing to achieve far transfers from PCT to sports performance may see far transfers, which can theoretically be achieved much more quickly, as a viable strategy to try to overcome the ‘slowness’ associated with the steady and systematic sequencing of near transfers. This is, however, unlikely to be a viable method given the limited supporting evidence for far transfers of PCT to sports performance.

Aside from its obvious message, this article also serves as a call to action for sport science to take a more collaborative and less siloed approach that encourages cross-talk between different disciplines. In doing so, it could be more attuned to the evidence that exists in other scientific disciplines, rather than merely reiterate what has already been done well elsewhere. This is by no means a call to cease research into the role played by PCT in the optimisation of sports performance. More and better research is needed to understand how PCT fits into athlete development. Instead, this article serves to encourage researchers to engage in cumulative science by looking beyond the borders of sport and exercise, in this case for example at the wealth of research on this topic in the psychological sciences. This is reiterated by Bayley [43], who argues that despite obvious flaws in scientific reasoning, some pseudoscientific (and practical) ideas and applications are very persistent. Additionally, this article is an encouragement of critical reflection over an acceptance of the status quo. If we continue to let those who shout the loudest (e.g. companies making questionable claims about the performance benefits of cognitive training) have the most prominent voice, we risk drowning out the critical voices of those who call out these claims. The aim of this article is not to be contrary, but instead to contribute to better scientific practices which ultimately lead to more reliable scientific implementations. Ultimately, we must decide if we are truly willing to reserve bold claims of discovery for findings that are transparently reported and withstand the scrutiny and verification that transparency invites, and thus ask ourselves if we want to be incredible, or credible [44].

## References

[1] Renshaw I, Davids K, Araújo D, Lucas A, Roberts WM, Newcombe DJ, Franks B. Evaluating weaknesses of “perceptual-cognitive training” and “brain training” methods in sport: an ecological dynamics critique. Front Psychol. 2019. 10.3389/fpsyg.2018.02468. 30719015 10.3389/fpsyg.2018.02468 PMC6348252

[2] Appelbaum LG, Erickson G. Sports vision training: a review of the state-of-the-art in digital training techniques. Int Rev Sport Exerc Psychol. 2018;11(1):160–89. 10.1080/1750984X.2016.1266376.

[3] Vater C, Gray R, Holcombe AO. A critical systematic review of the Neurotracker perceptual-cognitive training tool. Psychonom Bull Rev. 2021. 10.3758/s13423-021-01892-2. 10.3758/s13423-021-01892-2 PMC8500884 33821464

[4] Inns J, Petancevski EL, Novak AR, Fransen J. Decision-making assessments in youth team invasion game athletes: a systematic scoping review. Int J Sports Sci Coach. 2023;18(6):2360–81. 10.1177/17479541231185779.

[5] Walton CC, Keegan RJ, Martin M, Hallock H. The potential role for cognitive training in sport: more research needed. Front Psychol. 2018;9:1121. 10.3389/fpsyg.2018.01121. 30018585 10.3389/fpsyg.2018.01121 PMC6037849

[6] Hadlow SM, Panchuk D, Mann DL, Portus MR, Abernethy B. Modified perceptual training in sport: a new classification framework. J Sci Med Sport. 2018;21(9):950–8. 10.1016/j.jsams.2018.01.011. 29433921 10.1016/j.jsams.2018.01.011

[7] Choo L, Novak A, Impellizzeri FM, Porter C, Fransen J. Skill acquisition interventions for the learning of sports-related skills: a scoping review of randomised controlled trials. Psychol Sport Exerc. 2024. 10.1016/j.psychsport.2024.102615. 38401870 10.1016/j.psychsport.2024.102615

[8] Harris DJ, Wilson MR, Vine SJ. A systematic review of commercial cognitive training devices: implications for use in sport. Front Psychol. 2018. 10.3389/fpsyg.2018.00709. 29867674 10.3389/fpsyg.2018.00709 PMC5958310

[9] Sala G, Aksayli ND, Tatlidil KS, Tatsumi T, Gondo Y, Gobet F. Near and far transfer in cognitive training: a second-order meta-analysis. Collabra Psychol. 2019. 10.1525/collabra.203.

[10] Gobet F, Sala G. Cognitive training: a field in search of a phenomenon. Perspect Psychol Sci. 2023;18(1):125–41. 10.1177/17456916221091830. 35939827 10.1177/17456916221091830 PMC9903001

[11] Simons DJ, Boot WR, Charness N, Gathercole SE, Chabris CF, Hambrick DZ, Stine-Morrow EA. Do “brain-training” programs work? Psychol Sci Public Interest. 2016;17(3):103–86. 10.1177/1529100616661983. 27697851 10.1177/1529100616661983

[12] Laby DM, Appelbaum LG. Vision and on-field performance: a critical review of visual assessment and training studies with athletes. Optom Vis Sci. 2021;98(7):723–31. 10.1097/opx.0000000000001729. 34328451 10.1097/OPX.0000000000001729

[13] Romeas T, Guldner A, Faubert J. 3D-Multiple Object Tracking training task improves passing decision-making accuracy in soccer players. Psychol Sport Exerc. 2016;22:1–9. 10.1016/j.psychsport.2015.06.002.

[14] Liu S, Ferris LM, Hilbig S, Asamoa E, LaRue JL, Lyon D, et al. Dynamic vision training transfers positively to batting practice performance among collegiate baseball batters. Psychol Sport Exerc. 2020;51:101759. 10.1016/j.psychsport.2020.101759.

[15] Holton EF III. The flawed four-level evaluation model. Hum Resour Dev Q. 1996;7(1):5–21. 10.1002/hrdq.3920070103.

[16] Haberstroh T. How do Kawhi Leonard – and Steph Curry – train their brains? Strobe lights (yes, really). ESPN. 2016. https://www.espn.com.au/nba/story/_/id/18002545/kawhi-leonard-strobe-light-training-nba. Accessed 22 May 2024.

[17] Duke University. Strobe glasses improve hockey players’ performance. ScienceDaily. 2013. https://www.sciencedaily.com/releases/2013/12/131213201152.htm. Accessed 22 May 2024.

[18] Granger, J. Strobe sunglasses and tennis balls: Behind the Golden Knights’ Marc-Andre Fleury and his offseason workout. 2021. https://www.nytimes.com/athletic/2324749/2021/01/16/strobe-sunglasses-and-tennis-balls-behind-the-golden-knights-marc-andre-fleury-and-his-offseason-workout/. Accessed 22 May 2024.

[19] Cordy N. Sydney trial glasses that helped NBA star Steph Curry in bid to improve after poor start to season. The Daily Telegraph. 2017. https://www.dailytelegraph.com.au/sport/afl/teams/sydney/sydney-trial-glasses-that-helped-nba-star-steph-curry-in-bid-to-improve-after-poor-start-to-season/news-story/287ce8a77f64e1ef68ac8e82be30ca2b. Accessed 22 May 2024.

[20] Palmer T, Coutts AJ, Fransen J. An exploratory study on the effect of a four-week stroboscopic vision training program on soccer dribbling performance. Braz J Motor Behav. 2022;16(3):254–65. 10.2033/bjmb.v16i3.310.

[21] Schwab S, Memmert D. The impact of a sports vision training program in youth field hockey players. J Sports Sci Med. 2012;11(4):624. 24150071 PMC3763307

[22] Fransen J, Lovell TW, Bennett KJM, Deprez D, Deconinck FJ, Lenoir M, Coutts AJ. The influence of restricted visual feedback on dribbling performance in youth soccer players. Mot Control. 2017;21(2):158–67. 10.1123/mc.2015-0059. 10.1123/mc.2015-0059 27111662

[23] Beavan A, Hanke L, Spielmann J, Skorski S, Mayer J, Meyer T, Fransen J. The effect of stroboscopic vision on performance in a football specific assessment. Sci Med Football. 2021;5(4):317–22. 10.1080/24733938.2020.1862420. 10.1080/24733938.2020.1862420 35077302

[24] Appelbaum LG, Schroeder JE, Cain MS, Mitroff SR. Improved visual cognition through stroboscopic training. Front Psychol. 2011;2:276. 10.3389/fpsyg.2011.00276. 22059078 10.3389/fpsyg.2011.00276 PMC3203550

[25] Appelbaum LG, Cain MS, Schroeder JE, Darling EF, Mitroff SR. Stroboscopic visual training improves information encoding in short-term memory. Atten Percept Psychophys. 2012;74(8):1681–91. 10.3758/s13414-012-0344-6. 22810559 10.3758/s13414-012-0344-6

[26] Smith TQ, Mitroff SR. Stroboscopic training enhances anticipatory timing. Int J Exerc Sci. 2012;5(4):344. 27182391 10.70252/OTSW1297 PMC4738880

[27] Holliday J. Effect of stroboscopic vision training on dynamic visual acuity scores: Nike Vapor Strobe® Eyewear. 2013; Masters Thesis, on https://digitalcommons.usu.edu/gradreports/262/. Accessed 9 Nov 2023.

[28] Mitroff SR, Friesen P, Bennett D, Yoo H, Reichow AW. Enhancing ice hockey skills through stroboscopic visual training: a pilot study. Athlet Train Sports Health Care. 2013;5(6):261–4.

[29] Hülsdünker T, Rentz C, Ruhnow D, Käsbauer H, Strüder HK, Mierau A. The effect of 4-week stroboscopic training on visual function and sport-specific visuomotor performance in top-level badminton players. Int J Sports Physiol Perform. 2019;14(3):343–50. 10.1123/ijspp.2018-0302. 30160560 10.1123/ijspp.2018-0302

[30] Hülsdünker T, Gunasekara N, Mierau A. Short-and long-term stroboscopic training effects on visuomotor performance in elite youth sports. Part 1: reaction and behavior. Med Sci Sports Exerc. 2020;5:960–72. 10.1249/mss.0000000000002541. 10.1249/MSS.0000000000002541 33060548

[31] Carroll W, Fuller S, Lawrence JM, Osborne S, Stallcup R, Burch R, et al. Stroboscopic visual training for coaching practitioners: a comprehensive literature review. Int J Kinesiol Sports Sci. 2021;9(4):49–59. 10.7575/aiac.ijkss.v.9n.4p.49.

[32] Petancevski EL, Inns J, Fransen J, Impellizzeri FM. The effect of augmented feedback on the performance and learning of gross motor and sport-specific skills: a systematic review. Psychol Sport Exerc. 2022. 10.1016/j.psychsport.2022.102277.

[33] Ring C, Cooke A, Kavussanu M, McIntyre D, Masters R. Investigating the efficacy of neurofeedback training for expediting expertise and excellence in sport. Psychol Sport Exerc. 2015;16:118–27. 10.1016/j.psychsport.2014.08.005.

[34] Xiang MQ, Hou XH, Liao BG, Liao JW, Hu M. The effect of neurofeedback training for sport performance in athletes: a meta-analysis. Psychol Sport Exerc. 2018;36:114–22. 10.1016/j.psychsport.2018.02.004.

[35] Mirifar A, Beckmanm J, Ehrlenspiel F. Neurofeedback as supplementary training for optimizing athletes’ performance: a systematic review with implications for future research. Neurosci Biobehav Rev. 2017;75:419–32. 10.1016/j.neubiorev.2017.02.005. 28185873 10.1016/j.neubiorev.2017.02.005

[36] Laureys F, De Waelle S, Barendse MT, Lenoir M, Deconinck FJ. The factor structure of executive function in childhood and adolescence. Intelligence. 2020;90: 101600. 10.1016/j.intell.2021.101600.

[37] Beavan A, Spielmann J, Mayer J. Taking the first steps toward integrating testing and training cognitive abilities within high-performance athletes; insights from a professional German football club. Front Psychol. 2019;10:2773. 10.3389/fpsyg.2019.02773. 31920822 10.3389/fpsyg.2019.02773 PMC6923669

[38] Vestberg T, Gustafson R, Maurex L, Ingvar M, Petrovic P. Executive functions predict the success of top-soccer players. PLoS ONE. 2012;7(4): e34731. 10.1371/journal.pone.0034731. 22496850 10.1371/journal.pone.0034731 PMC3319604

[39] Beavan A, Spielmann J, Mayer J, Skorski S, Meyer T, Fransen J. The rise and fall of executive functions in high-level football players. Psychol Sport Exerc. 2020;2020(49):101677. 10.1016/j.psychsport.2020.101677. 10.1123/jsep.2019-0312 32711397

[40] Beavan A, Chin V, Ryan LM, Spielmann J, Mayer J, Skorski S, et al. A longitudinal analysis of the executive functions in high-level soccer players. J Sport Exerc Psychol. 2020;42(5):349–57. 10.1123/jsep.2019-0312. 32711397 10.1123/jsep.2019-0312

[41] Beavan A, Spielmann J, Ehmann P, Mayer J. The development of executive functions in high-level female soccer players. Percept Motor Skills. 2022. 10.1177/00315125221096989. 35521695 10.1177/00315125221096989

[42] Scharfen HE, Memmert D. Cognitive training in elite soccer players: evidence of narrow, but not broad transfer to visual and executive function. German J Exerc Sport Res. 2021;51(2):135–45. 10.1007/s12662-020-00699-y.

[43] Bayley, R. Science, pseudoscience and exercise neuroscience. Physical activity and educational achievement: insights from exercise neuroscience. 2017;335

[44] Vazire S. Do we want to be credible or incredible? 2019 APS Observer, 33. https://www.psychologicalscience.org/observer/do-we-want-to-be-credible-or-incredible. Accessed 2 Aug 2022.

